# Mutant ATRX: pathogenesis of ATRX syndrome and cancer

**DOI:** 10.3389/fmolb.2024.1434398

**Published:** 2024-10-16

**Authors:** Kejia Yuan, Yan Tang, Zexian Ding, Lei Peng, Jinghua Zeng, Huaying Wu, Qi Yi

**Affiliations:** Key Laboratory of Model Animals and Stem Cell Biology, Hunan Normal University School of Medicine, Changsha, Hunan, China

**Keywords:** ATRX, ATRX syndrome, replication stress, ALT, transcriptional regulation, tumorigenesis

## Abstract

The transcriptional regulator ATRX, a genetic factor, is associated with a range of disabilities, including intellectual, hematopoietic, skeletal, facial, and urogenital disabilities. ATRX mutations substantially contribute to the pathogenesis of ATRX syndrome and are frequently detected in gliomas and many other cancers. These mutations disrupt the organization, subcellular localization, and transcriptional activity of ATRX, leading to chromosomal instability and affecting interactions with key regulatory proteins such as DAXX, EZH2, and TERRA. ATRX also functions as a transcriptional regulator involved in the pathogenesis of neuronal disorders and various diseases. In conclusion, ATRX is a central protein whose abnormalities lead to multiple diseases.

## 1 Introduction

The ATRX gene was first identified as a pathogenic gene for a rare hereditary disease that causes intellectual disability and α-thalassemia more than two decades ago (Weatherall et al., 1981; Gibbons et al., 2008; Watson et al., 2015; León and Harley, 2021). ATRX belongs to the switch/sucrose nonfermentable (SWI-SNF) protein clan and functions as a chromatin remodeler, many of which can slide, modify, or remove histones *in vitro* (Stayton et al., 1994; Picketts et al., 1996; Argentaro et al., 2007; Clynes et al., 2013). Recent research indicates that ATRX is involved in various biological activities, such as nucleosome remodeling (Gibbons et al., 1995; Xue et al., 2003; Bérubé et al., 2008), transcriptional regulation (Li et al., 2016; Chu et al., 2017; Schenkel et al., 2017; Truch et al., 2022), recombination (Bérubé et al., 2002; Clynes et al., 2015; Gramatzki et al., 2016; Teng et al., 2021; Liu and Malkova, 2022), DNA repair (Nasmyth and Haering, 2009; Clynes et al., 2015; Han et al., 2020), and tumorigenesis (Picketts et al., 1996; Danussi et al., 2018; Ohba et al., 2020; Gulve et al., 2022; Hackeng et al., 2022).

ATRX syndrome includes five main typical clinical manifestations: facial dysmorphism, hypotonia, skeletal, genitourinary system and hematopoietic abnormalities. Mental retardation, which is a consistent clinical characteristic in all ATRX syndrome patients, is the most basic clinical manifestation of the ATRX syndrome diagnosis (León and Harley, 2021). ATRX syndrome is usually asymptomatic in women due to significant skewing of X chromosome offset inactivation, while the symptoms are more obvious in men due to the absence of alleles (Wada et al., 2005). ATRX syndrome is primarily caused by ATRX mutations and characterized by ATRX deletion or missense mutations (Masliah-Planchon et al., 2018; Fan et al., 2019). This syndrome arises from several pathways, including neuronal differentiation, telomere stability, DNA damage response (DDR) pathways, and altered expression and structure of ATRX-interacting proteins (Hong et al., 2009; De La Fuente et al., 2011; Lacoste et al., 2014; Li et al., 2016; Garbarino et al., 2021). In addition, studies have shown that patients with ATRX syndrome have a genetic predisposition to osteosarcoma, which offers a novel avenue for diagnosing ATRX syndrome as a nonclassical phenotype of ATRX syndrome (Ji et al., 2017).

ATRX mutations are identified in a range of malignant tumors, such as neuroblastoma, pancreatic neuroendocrine tumors, and osteosarcoma. Up to 25% of gliomas exhibit ATRX mutations, with specific percentages including 67% of WHO grade II astrocytomas, 73% of WHO grade III astrocytomas, and 57% of secondary glioblastomas (Olar and Sulman, 2015).

In approximately 10%–15% of tumors, telomere length is maintained via alternative lengthening of telomeres (ALT) rather than telomerase activation (Shay and Wright, 2019). The ALT pathway is activated by telomere replication stress and DNA damage and relies on break-induced replication (BIR) for complete DNA replication. This process promotes the formation of ALT-associated PML (APB) bodies, which in turn trigger BIR to elongate telomeres (Dilley et al., 2016). In the past decade, high-throughput exon and whole-genome sequencing has shown that mutations in the histone protein ATRX and its partner DAXX are associated with an increased incidence of ALT activation in various cancer types (Heaphy et al., 2011; Schwartzentruber et al., 2012; Ceccarelli et al., 2016). ATRX or DAXX mutations in human tumors are mutually exclusive to TERT promoter mutations (Killela et al., 2013; Brat et al., 2015; Eckel-Passow et al., 2015; Barthel et al., 2017). A survey of 22 immortalized human ALT cell lines indicated that approximately 90% of the cells exhibited abnormal expression of the ATRX and/or DAXX proteins (Lovejoy et al., 2012), confirming the association between the ATRX/DAXX status and ALT activation.

Due to the crucial role of telomere length maintenance in cell perpetuation and tumorigenesis, the strong correlation between ATRX deficiency and ALT activation suggests that ATRX might suppress tumors by directly inhibiting ALT (Pickett and Reddel, 2015; Maciejowski and de Lange, 2017). Restoring ATRX expression in ATRX-negative ALT cell lines significantly reduced ALT-associated phenotypes (Clynes et al., 2015; Napier et al., 2015). ATRX is proposed to suppress ALT through multiple nuclear functions, including telomere H3.3 loading (Wong et al., 2010), telomere condensation resolution (Ramamoorthy and Smith, 2015), protection of DNA replication from stress or G-quadruplex formation (Law et al., 2010; Leung et al., 2013; Clynes et al., 2015) and telomere R-loop repression (Nguyen et al., 2017).

ATRX mutations are also associated with IDH (isocitrate dehydrogenase)-mutant tumors, which have a high incidence of ATRX mutations (Kannan et al., 2012). ATRX loss, attributed to mutations, deletions, or gene fusions, is linked to other distinct molecular alterations, such as the ALT phenotype (Nandakumar et al., 2017).

This paper highlights the various abnormalities associated with the ATRX protein, from its structure, interactive proteins, and abnormal expression to ATRX as a transcription factor affecting protein expression. In addition, the role of ATRX in tumorigenesis and its mechanism is described to comprehend its role in normal biology and tumors and explore potential research directions in pathogenesis of ATRX syndrome and cancer. Gaining insight into ATRX’s role in cancer will help develop more effective and targeted anti-cancer therapies.

## 2 Main text

In order to explore the role of ATRX in ATRX syndrome and tumors, we introduced the structural mutations of ATRX, abnormal interacting proteins and transcriptional regulation, the effect of deletion on disease, the effect of mutations on ALT formation, tumor immunity, post-translational modification. We also summarized the effects of various ATRX mutation types on the development of disease, and investigate future directions for ATRX as a potential therapeutic target or prognostic biomarker.

### 2.1 Structural mutations of ATRX

ATRX is composed of two separate regions: a cysteine-rich ATRX-DNMT3-DNMT3L (ADD) motif domain at the N-terminus and an ATP-dependent helicase domain at the C-terminus (Olar and Sulman, 2015). The ADD domain features two distinctive fingers, a GATA-like finger at the N-terminal end and a PHD finger at the C-terminal end, which are involved in chromatin localization (Gibbons et al., 2008; Iwase et al., 2011). Previous research has shown that ATRX is attracted to pericentromeric heterochromatin (PCH) via its binding to histone H3 trimethylated on lysine 9 (H3K9me3) through the ADD domain but is inhibited by histone H3 trimethylated on lysine 4 (H3K4me3) (Iwase et al., 2011). The majority of missense mutations leading to ATRX syndrome occur within the ADD domains in ATRX patients (Gibbons et al., 2008; Iwase et al., 2011). Mutations in the ADD domain caused a marked reduction in binding to H3K9me3, consequently disrupting the subnuclear localization of ATRX. These aberrations are closely correlated with severe impairments in neuronal differentiation, psychomotor function, and urogenital development (Badens et al., 2006; Gibbons et al., 2008; Dhayalan et al., 2011; Palaniappan and Ramalingam, 2017; Bieluszewska et al., 2022).

**FIGURE 1:**
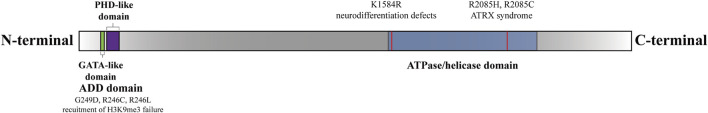
Functional domains and sites of the ATRX (Gibbons et al., 2008). There are two functional domains of ATRX: The N-terminal ADD-domain and the C-terminal ATPase/helicase-domain. The ADD domain bears the GATA-like finger followed by a PHD finger. ADD-domain is required for targeting pericentromeric heterochromatin (PCH) by binding to H3K9me3. The mutations G249D, R246C, and R246L hinder ATRX’s capability to bind to H3K9me3 (Dhayalan et al., 2011). The K1584R mutant induces specific neurodevelopmental abnormalities (Bieluszewska et al., 2022). The R2085H and R2085C mutants are potential genetic triggers of ATRX syndrome (Lacoste et al., 2014).

The C-terminus of ATRX harbors a septet of helicase domains responsible for ATPase and helicase functions. The C-terminal helicase domain has been implicated in both ATRX syndrome and cancer (Gibbons et al., 2008; Clynes et al., 2013; Bieluszewska et al., 2022). Importantly, ATRX syndrome is attributed to recurrent substitutions in a highly conserved residue, residue 2,085, within the helicase domain of ATRX (Lacoste et al., 2014). Additionally, the K1584R mutation induces mouse embryonic stem cells (mESCs) to differentiate into neural progenitor cells (NPCs) while disrupting glucose metabolic pathways, resulting in distinct neurodevelopmental defects (Bieluszewska et al., 2022). The K1584R mutant is unable to hydrolyze ATP, thus increasing binding to nucleic acids, which results in increased enrichment of ATRX at target sites but decreased localization to pericentromeres (Mitson et al., 2011; Sarma et al., 2014; Bieluszewska et al., 2022) (Figure 1). Mutations associated with ATRX syndrome primarily manifest as missense mutations in the regions described above. However, the precise mechanisms underlying ATRX syndrome resulting from mutations in distinct regions are inadequately explored. Further investigation is warranted to elucidate how these mutations impact the subnuclear localization of ATRX, the pathological consequences of aberrant ATRX localization, and the differential effects of mutations across various regions on the development of intellectual impairment.

**FIGURE 2:**
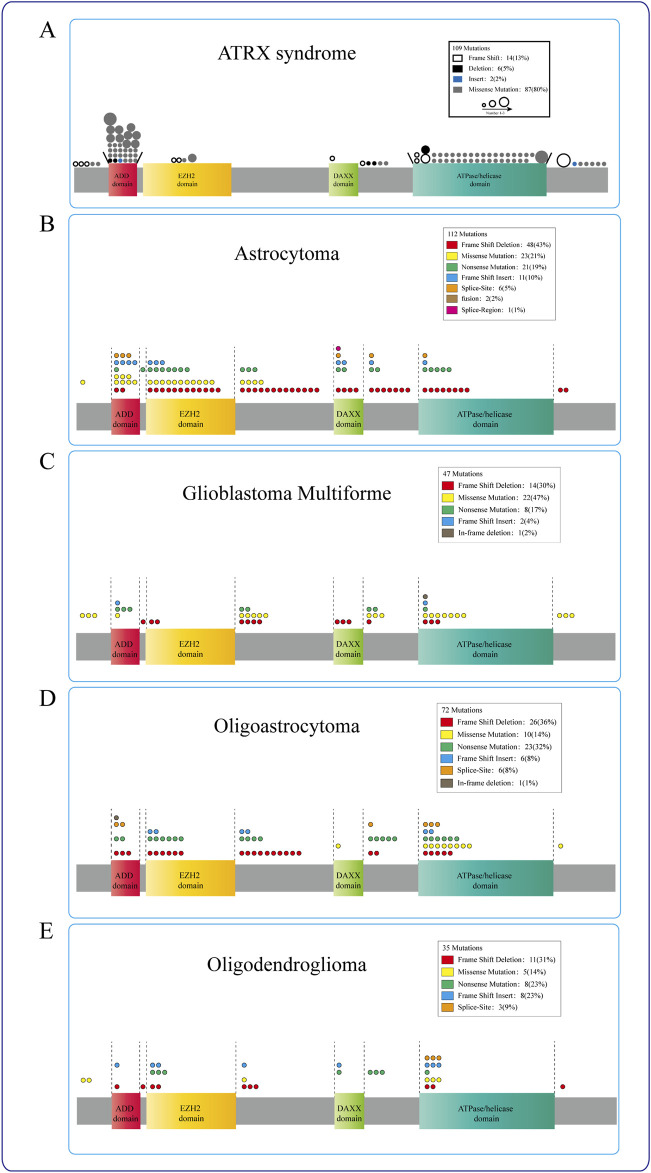
ATRX mutations in diseases. **(A)** ATRX patient mutations (circle) are predominantly found in either the ADD or the C-terminal ATP-dependent helicase domain, different circles indicate different ways of mutation, and the circle size represents the mutation frequency at the same locus. **(B–E)** The mutation sites of ATRX in Astrocytoma, Glioblastoma multiforme, Oligoastrocytoma, and Oligodendroglioma were mostly concentrated in the ATRX functional domain. Different colors represent different mutation modes, and the number of circles indicates the frequency with which this mutation mode occurs in this region.

In the cBioPortal datasets, 884 ATRX mutations have been identified in various cancers. Among these mutations, 267 (30.2%) are associated with glioblastoma multiforme (GBM), oligoastrocytoma, oligodendroglioma, low-grade glioma (NOS), and astrocytoma. In the TCGA glioma datasets, approximately 21% of the samples exhibited ATRX mutations, with truncating mutations and deletions most commonly occurring. The presence of ATRX mutations is linked to various pivotal molecular occurrences (Xie et al., 2019). However, the precise roles of missense, truncating, inframe, splice, and fusion variants in these tumors have not been well investigated. These changes, particularly truncating mutations, occur throughout multiple protein domains and result in decreased protein expression. ATRX mutation and aberrant p53 expression are frequently present in IDH-mutant astrocytomas, which exhibit chromosomal instability and the ALT phenotype, eventually progressing to glioma (Heaphy et al., 2011; Marinoni et al., 2014; Ohba et al., 2020) (Figure 2).

### 2.2 Interacting proteins

Numerous proteins interact with ATRX in cells, collectively participating in various cellular processes. These proteins mediate H3.3 deposition, DNA methylation, the regulation of heterochromatin epigenetics and DNA transcription. These proteins facilitate the localization of ATRX to PCH, aiding chromosome cohesion during mitosis, and contribute to DNA double-strand break repair, DNA replication, and telomere maintenance. Mutations in these interacting proteins can disrupt the function of ATRX, causing unusual transcription and the onset of the ALT phenotype. This phenotype is strongly linked to the manifestation of ATRX syndrome and a range of tumors (Table 1).

**TABLE 1:** ATRX interacting proteins, relative binding domains, function, and results of mutation.

Interacting protein	Interacting domain	Function	Results of mutation
DAXX	DAXX binding domain 1189-1326aa	H3.3 deposition	Chromosomal instability
MeCP2	MeCP2 interacting region 1915-2492aa	DNA methylation and PCH formation	Less co-localization at PCH; Less expression in terminally differentiated neurons
EZH2	EZH2 binding domain 321-734aa	DNA methylation	α-thalassaemia; α-globin reductive expression; Apoptosis
TERRA	Probably RBR domain	Telomeric maintenance Regulations of telomeres localizations Gene expression	ALT; G4 formation
MCM	Unknown	DNA replication	DSBs; Replicative stress
MRN	Unknown	Inhibition of ALT	HR; ALT
RAD21	Unknown	Mending DSBs	Replicative stress; DSBs
SMC1	Unknown	Regulation of mitotic chromosome cohesion	Replicative stress; DSBs
SMC3	Unknown	Regulation of mitotic chromosome cohesion	Replicative stress; DSBs
SA1	Unknown	Regulation of mitotic chromosome cohesion Repair of DSBs in G2	Replicative stress; DSBs
SA2	Unknown	Regulation of mitotic chromosome cohesion Repair of DSBs in G2	Replicative stress; DSBs
TPP1	Unknown	Mending DSBs	Replicative stress; DSBs

#### 2.2.1 DAXX

Initially, identified as a mediator of Fas-induced apoptosis, death domain-associated protein (DAXX) was suggested to directly interact with Fas. ATRX interacts with DAXX, acting as a histone chaperone to form a chromatin remodeling complex. This complex facilitates the inclusion of the replication-independent histone variant H3.3 in transcriptionally repressed regions such as PCH, telomeres, and other repetitive elements, which are crucial for maintaining chromatin stability (Goldberg et al., 2010; Bogolyubova and Bogolyubov, 2021). These regions are often enriched with histone marks such as H3K9me3, H4K20me3, and DNA methylation (Yang et al., 1997; Xue et al., 2003; Tang et al., 2004; Goldberg et al., 2010; Law et al., 2010; Lewis et al., 2010; Voon et al., 2015). Unlike histone H3, histone H3.3 is not involved in DNA synthesis in the S phase (Goldberg et al., 2010). H3.3 serves as a “replacement” histone in genomic regions lacking histones during phases outside of S phase, restoring chromatin status (Wu et al., 1982; Henikoff et al., 2004).

DAXX specifically binds to H3.3, acting as a chaperone, while ATRX directs the DAXX-mediated incorporation of H3.3 into H3K9me3-enriched chromatin regions via its ADD domain (Iwase et al., 2011). The histone variant H3.3 is present in both transcribed genes and constitutive heterochromatin, but its deposition in these regions involves distinct mechanisms (Goldberg et al., 2010; Wong et al., 2010). When ATRX/DAXX is lacking, histone cell cycle regulator (HIRA) can substitute for the deposition of H3.3 in telomeric heterochromatin (Ahmad and Henikoff, 2002; Tagami et al., 2004). Unlike ATRX/DAXX, HIRA specifically deposits H3.3 in transcriptionally active genome regions, like gene bodies, promoters, and enhancers (Tagami et al., 2004; Wirbelauer et al., 2005; Goldberg et al., 2010).

When ATRX is lost, heterochromatin regions fail to deposit H3.3, causing DNA damage, telomeric end fusions, and genomic instability, ultimately affecting the transcription of tandem repeats and telomere functions (Haase et al., 2018). Additionally, ATRX colocalizes with SETDB1, an H3K9 histone methyltransferase. The ATRX and DAXX complex maintains H3K9me3 at transcriptional sites, but when one component of the complex or H3.3 is depleted, H3K9me3 is reduced among repetitive regions.

ATRX and DAXX act as tumor suppressor proteins and are typically mutated in cells exhibiting ALT-like characteristic gliomas (Lovejoy et al., 2012; Gulve et al., 2022; Clatterbuck Soper and Meltzer, 2023). These mutations lead to the downregulation of the p53 pathway and a diminished response to DNA damage (Gulve et al., 2022).

#### 2.2.2 PML-NBs

The ATRX and DAXX complex is enriched in promyelocytic leukemia nuclear bodies (PML-NBs) (Ishov et al., 1999; Xue et al., 2003). PML-NBs harbor a multitude of proteins, consisting of the ATRX, DAXX and H3.3 complex, as well as SUMO1/2/3 (Ishov et al., 1999; Delbarre et al., 2013). PML, the primary component of PML-NBs, aids H3.3 chromatin assembly during DNA replication and inhibits DAXX-mediated assembly by recruitment to H3.3 in heterochromatin in the S phase. PML is also a crucial site for mutation and dysregulation that affects the epigenetic inheritance of heterochromatin and induces a variety of diseases (Shastrula et al., 2019).

**FIGURE 3:**
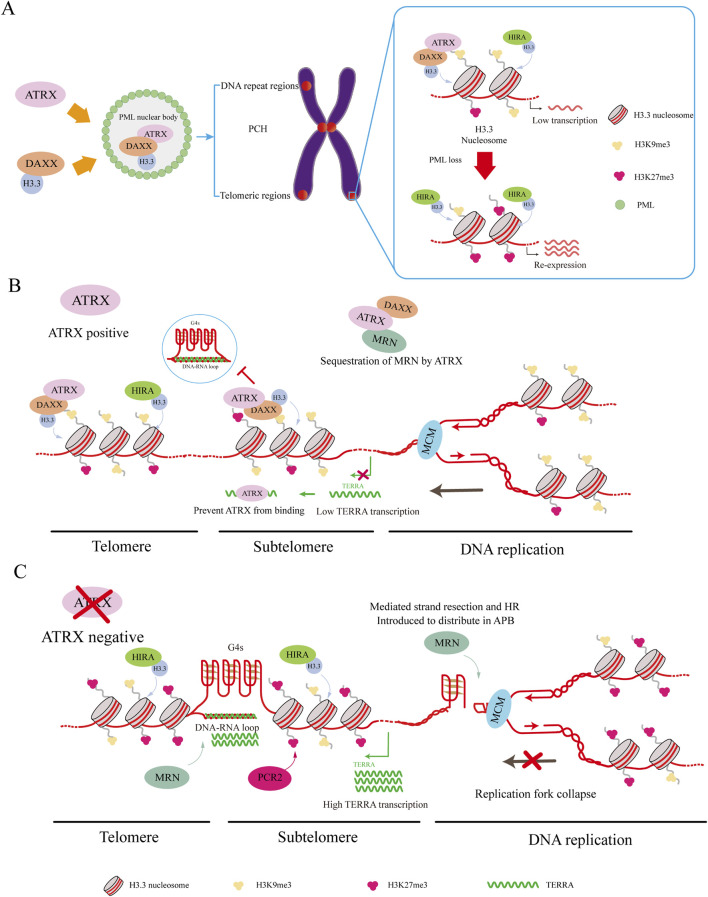
ATRX interacting proteins. **(A)** DAXX binds to H3.3 and carries it to PML-NBs for complex assembly. This complex targets H3K9me3, leading to the deposition of H3.3 in regions such as PCH, telomeric regions, and other DNA repeat regions, resulting in silent gene expression. HIRA can also deposit H3.3 in these regions. Loss of PML-NBs causes loss of H3K9me3 and increased H3K27me3 misdeposition, which leads to re-expression of silenced genes. **(B)** ATRX maintains telomeric and subtelomeric chromatin integrity while aiding DNA replication. ATRX facilitates the relocation of the MRN complex from telomeric DNA. Additionally, it resolves G4 DNA structures encountered by the replicative MCM helicase complex and deposits H3.3 to promote MCM progression. Meanwhile, ATRX binds to TERRA to suppress its expression and down-regulate the co-localized genes. **(C)** In ATRX deficiency, elevated TERRA expression leads to the formation of DNA: RNA hybrids called R-loops. These R-loops hinder telomere replication. Meanwhile, G4 formation causes replication fork collapse, redirecting MRN to the APB site. These promote the occurrence of ALT and recombination-mediated processes at the telomere.

Two methods have been proposed to connect ATRX/DAXX/H3.3 deposition with PML-NBs. In the traditional method, DAXX is drawn to PML bodies by its SUMO-interacting motif (SIM) domains (Lin et al., 2006; Santiago et al., 2009), while the localization of ATRX to PML bodies hinges on its interaction with DAXX (Tang et al., 2004). PML bodies store soluble H3.3-H4 heterodimers, after which ATRX and DAXX are deposited (Delbarre et al., 2013). Recent research has indicated that PML-associated domains (PADs) are extensive heterochromatic regions where ATRX/DAXX/H3.3 complexes are deposited by PML-NBs (Delbarre et al., 2017). Without PML, the ATRX and DAXX complex lacks H3.3 deposition in these regions (Delbarre et al., 2017). At the same time, the epigenetic signature of the pad is altered, with increased H3K27me3 and loss of H3K9me3 (Delbarre et al., 2017). Both H3K9me3 and H3K27me3 are involved in maintaining genomic stability, and this genetic shift serves as a compensatory mechanism when ATRX cannot target PADs (Delbarre et al., 2017). However, increasing H3K27me3 to maintain heterochromatin in PADs might depend on the ability of ATRX to recruit polycomb repressive complex 2 (PCR2) complexes to heterochromatin (Sarma et al., 2014; Delbarre et al., 2017) (Figure 3A).

This discovery underscores the unique connection between ATRX/DAXX and PML, elucidating why the loss of PML leads to alterations in the epigenetic marks of PADs. Unlike ATRX, HIRA maintains its capacity for H3.3 deposition into PADs without elevating H3K27me3, supporting a mechanism to fill gaps and maintain chromatin stability (Ray-Gallet et al., 2011; Delbarre et al., 2017). The shift of H3K9me3 to H3K27me3 can preserve heterochromatin, but changes in some regions can lead to the misdeposition of H3K27me3, causing impoverishment of H3K27me3 in some regions. As a result, some tumors exhibit re-expression of silenced genes when ATRX is depleted (Sarma et al., 2014; Danussi et al., 2018; Oppel et al., 2019; Han et al., 2020) (Figure 3A).

#### 2.2.3 MeCP2

Through epigenetic imprinting and chromatin condensation, methyl-CpG binding protein 2 (MeCP2) modulates gene expression and functions as a transcriptional regulator (Haase et al., 2022). Functioning as a transcriptional silencing factor, MeCP2 promotes the attraction of histone deacetylases and comodulators by binding specifically to methylated DNA (Marano et al., 2019). In mature neurons, MeCP2 and a major satellite forwards transcript target ATRX to the PCH and regulate its expression (Marano et al., 2019). Pathological alterations leading to intellectual disability and Rett syndrome (RTT) may arise from point mutations in the MeCP2 gene, which disrupt the MeCP2-ATRX interaction (Nan et al., 2007; Gibbons et al., 2008). Similar results have been predicted for mice (Nan et al., 2007; Kernohan et al., 2010; Kernohan et al., 2014; Haase et al., 2022). Kristin’s study found that ATRX collaborates with MeCP2 to repress a specific group of imprinted genes in the postnatal mouse brain. Mutated MeCP2 interferes with the brain’s repression of imprinted genes, increasing the cognitive deficits associated with RTT and ATRX syndromes (Nan et al., 2007; Kernohan et al., 2010).

#### 2.2.4 EZH2

EZH2 plays a pivotal role in catalyzing H3K27me3 modification, which inhibits target gene transcription by causing chromatin condensation. ATRX interacts with EZH2 through its SET domain to sequester EZH2 to limit the repressive function of EZH2 (Cardoso et al., 1998; Zeng et al., 2022). Prior research has demonstrated a correlation between EZH2 and VAV1, a proto-oncogene, which forms the VAV1-EZH2-ATRX complex. This complex plays a crucial role in hematopoiesis and has been associated with α-thalassemia. The helicase activity of ATRX opposes EZH2 repression, thus promoting α-globin gene expression. Therefore, ATRX deficiency suppresses α-globin gene expression, contributing to disease development (Ye and Worman, 1996; Cardoso et al., 1998; Bieluszewska et al., 2022). Furthermore, inhibiting EZH2 in neuroblastoma (NB) patients upregulates genes related to neuronal maturation, inducing apoptosis in ATRX in-frame fusion (IFF) NB cells (Qadeer et al., 2019). Temozolomide (TMZ) serves as the primary chemotherapeutic agent for glioma treatment. The chemoresistance observed in gliomas is related to ATRX. Compared to parental cells, TMZ-resistant glioma cells expressed higher levels of ATRX and were enriched in EZH2 (Han et al., 2020). This promotes DNA damage repair mechanisms and facilitates the proliferation of glioma cells (Han et al., 2020).

#### 2.2.5 TERRA

ATRX acts as a chromatin remodeling element, regulating gene expression in regions of DNA called G-quadruplexes (G4s), which are found in G-rich repetitive sequences of DNA, including telomeric repeats (Law et al., 2010; Yeung et al., 2013). These G4 structures may lead to DNA damage, acting as substrates for homologous recombination (HR) and inducing a process called ALT during DNA replication (Gramatzki et al., 2016; Teng et al., 2021). ATRX is supposed to prevent this by resolving G4 motifs preceding the replication fork; however, it lacks the ability to resolve these structures on its own (Law et al., 2010; Watson et al., 2013; Clynes et al., 2014; Clynes et al., 2015; Wang et al., 2019). Another molecule that maintains telomeres is the long noncoding RNA telomeric repeat-containing RNA (TERRA), which is composed of the repetitive RNA sequence UUAGGG (Azzalin et al., 2007; Schoeftner and Blasco, 2008). Its synthesis occurs from subtelomeric regions towards telomeric ends (Azzalin et al., 2007; Nergadze et al., 2009). As TERRA invades double-stranded DNA, it forms an RNA‒DNA hybrid (R-loop) at its target site, which aids in the generation of DNA G4s (Tsai et al., 2022).

TERRA and ATRX share hundreds of genes, and their competition for binding at the telomeric region suppresses ATRX localization and maintains telomeric stability (Chu et al., 2017). TERRA can also regulate gene expression in two ways: cis and trans. In cis, ATRX, which is enriched in subtelomeric genes, contributes to H3K9me3-mediated chromatin condensation and downregulates gene expression after TERRA deletion. In trans, TERRA promotes the formation of G4 structures at transcription start sites (TSSs) and prevents ATRX from binding, which leads to the upregulation of gene expression. When TERRA is deleted, the level of ATRX near the TSS increases, resulting in G4 DNA unwinding and gene repression (Tsai et al., 2022).

TERRA strongly binds to DNA via H3K27me3, which is modified by EZH2, while ATRX is associated with H3K9me3 (Sarma et al., 2014; Clynes et al., 2015). When TERRA is deleted, ATRX and H3K9me3 occupy subtelomere regions more frequently, which leads to the downregulation of certain genes that are targeted by both TERRA and ATRX, including Edr1, Mid1, Asmt, Fyco1, and Lphn2 (Chu et al., 2017). Knocking down the ATRX protein by siRNA largely leads to the upregulation of the genes mentioned above (Chu et al., 2017).

According to previous studies, R-loops and G4s can inhibit DNA replication, increase telomere replication stress, and initiate the ALT pathway. However, studies have also demonstrated that ATRX can repress the formation of R-loops both *in vivo* and *in vitro* (Tsai et al., 2022; Yan et al., 2022). By inhibiting R-loop formation, ATRX prevents G-rich single-stranded DNA extrusion, preventing G4 formation (Tsai et al., 2022) (Figures 3B, C).

#### 2.2.6 Cohesion

Canonical cohesion is a ring-shaped complex consisting of structural maintenance of chromosomes 1 and 3 (SMC1, SMC3), the Rad21 cohesion complex (RAD21), and either stromal antigen 1 or 2 (SA1 or SA2) (Liu et al., 2023). This complex is crucial for accurate sister chromatid segregation during mitosis and double-strand break (DSB) repair in G2 (Nasmyth and Haering, 2005; Hirano, 2006; Onn et al., 2008; Peters et al., 2008; Nasmyth and Haering, 2009). Among these proteins, ATRX and SA1 can repair DSBs in a similar way by collaborating with the ACD shelterin complex subunit and telomerase recruitment factor (TPP1) to promote the cohesion of telomeres and inhibit nonallelic telomere associations. In addition, SA1 and SA2 cooperate with ATRX-DAXX, which facilitates telomeric DSB repair (Lovejoy et al., 2020). Therefore, in many telomerase-deficient cancers, ATRX inhibits the ALT pathway by promoting cohesion functions in both sister chromatid segregation and telomeric repair of DSBs during G2. However, the detailed mechanisms underlying the interactions between these components warrant further investigation.

#### 2.2.7 MRN

The MRE11-RAD50-NBS1 (MRN) complex is crucial for maintaining the stability of the genome and supporting DNA replication. This complex serves as a DNA damage sensor and helps restart replication forks and repair DNA damage (Clynes et al., 2015). Moreover, the ATRX complex aids in the removal of DNA ends from 5′ to 3′, generating a 3′overhang crucial for subsequent strand invasion (Bérubé et al., 2005; Clynes et al., 2015). ATRX presence reorganizes the MRN complex, relocating it from telomeric DNA and PML nuclear bodies (Clynes et al., 2015). If ATRX is not expressed, the MRN complex is reassigned to the APB site, where it may promote HR (Bérubé et al., 2002; Clynes et al., 2015) (Figures 3B, C).

#### 2.2.8 MCM

The minichromosome maintenance complex (MCM) is a DNA helicase that is essential to initiate DNA replication and elongation (Wu et al., 2018). Studies indicate a connection between ATRX and MCM, and ATRX can utilize its helicase activity to resolve G4 DNA to promote MCM progression in the presence of G4 DNA. At the same time, DAXX mediates variant H3.3 deposition to contain the G4 region in closed heterochromatin, reducing chromatin accessibility and preventing chromosome instability (Teng et al., 2021). Cells are protected from replication stress caused by G4 structures by these activities occurring in the upper reaches of heterochromatin formation mediated by ESET (Figures 3B, C).

### 2.3 ATRX acts as a transcriptional factor

α-Thalassaemia is a phenotype of ATRX syndrome characterized by decreased expression of α-globin (Truch et al., 2022). ATRX acts as a transcription factor pivotal in regulating α-globin expression. Therefore, mutations in ATRX are significant contributors to alpha thalassemia myelodysplastic syndrome (ATMDS), which is a severe form of α-thalassemia (Gibbons et al., 2003; Steensma et al., 2005; Nguyen et al., 2023).

The variable number of tandem repeats (VNTR) sequence, with the potential G-quadruplex-forming sequence CGC(GGGGCGGGG) n, contributes to the downregulation of α-globin expression (Li et al., 2016). ATRX and DAXX bind collaboratively to the VNTR sequence in the α-globin gene cluster promoter, resolving G4 and promoting the transcription of α-globin (Li et al., 2016). Additionally, using its ADD domain, ATRX identifies H3K9me3-containing nucleosomes (H3.1 and H3.2) and swaps macroH2A for H3.3 (via DAXX) at the α-globin locus. This process promotes a switch towards open chromatin and transcription of α-globin (Ratnakumar and Bernstein, 2013). For patients with ATRX syndrome, mutations or the absence of ATRX can alter VNTR sequence lengths, increasing the presence of variant macroH2A at the α-globin locus and leading to transcriptional repression of the hemoglobin α (HBA) gene at the same locus (Pan and Sawalha, 2009; Ratnakumar et al., 2012; Truch et al., 2022; Aguilera and López-Contreras, 2023).

Moreover, ATRX-mediated regulation of transcription with TERRA at telomeres has been described above, but the specific protein regulating transcription by ATRX is closely related to tumorigenesis and deserves further study. Overall, ATRX, which functions as a transcription factor, regulates the transcription of downstream proteins by deregulating aberrant DNA structures that affect transcription, changing histone deposition, and competing sites for transcription. More modes of regulation remain to be investigated.

### 2.4 Aberrant expression of ATRX

Brain and neuronal development rely on ATRX, and its aberrant expression contributes to intellectual disability (Bérubé et al., 2005; Ritchie et al., 2008; Medina et al., 2009). Several developmental defects and strong apoptosis were observed in *Drosophila* neurons when ATRX was inhibited, resulting in caspase-dependent apoptosis via the JNK and dFOXO pathways (Hong et al., 2009). ATRX syndrome is associated with the overexpression of ATRX in the nucleus. Excessive levels of ATRX have been shown to induce growth impairment, neural tube malformations, and heightened embryonic death in transgenic mice (Bérubé et al., 2002). Furthermore, the absence of the ATRX protein can exacerbate neuronal loss, culminating in severe intellectual disability in humans (Bérubé et al., 2005). ATRX deficiency can trigger p53-dependent neuronal apoptosis. Despite the use of ATRX-p53 double knockout mice to rescue embryonic cortex cell death and basal ganglia, this model cannot address all aspects of ATRX deficiency in the developing forebrain (Seah et al., 2008). Postnatal forebrain excitatory neurons lacking ATRX (ATRX-cKO) are less able to transmit synaptic signals and lack plasticity in the hippocampal formation (Gugustea et al., 2020).

ATRX/DAXX/H3.3 are crucial for preserving telomere structural integrity (Ahmad and Henikoff, 2002; Heaphy et al., 2011). Loss of ATRX impairs nucleosome histone H3.3 loading to human telomeres, reducing nucleosome assembly during telomere replication and causing a progressive increase in the length of unbound DNA segments between nucleosomes (Clynes et al., 2014; Koschmann et al., 2016; Li et al., 2019). When these DNA segments reach a certain length, they break or form secondary structures such as G4, impeding telomere replication and provoking a DNA damage response. Retaining telomeric DNA damage can hamper cell growth, accelerate aging, and impede the proliferation of cells (Li et al., 2019). ATRX-deficient tumors always present with ALT (Lovejoy et al., 2012; Amorim et al., 2016; Koschmann et al., 2016). However, the absence of ATRX leads to the concurrent stabilization of G4 structures, impeding these repair pathways, thereby exacerbating DNA damage and precipitating cell death (Wang et al., 2019).

### 2.5 ATRX mutations in cancer

**FIGURE 4:**
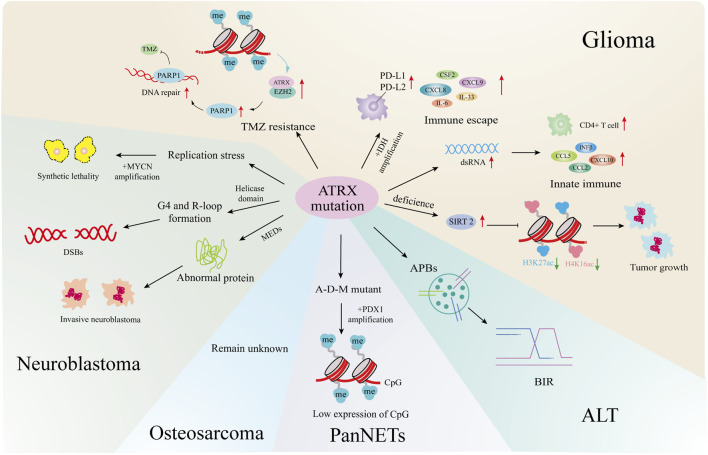
ATRX mutations and cancers. In gliomas, ATRX mutations is involved in the development of ALT, the formation of TMZ resistant strains, the changes of the immune microenvironment, and affects tumor growth and senescence through epigenetics. In neuroblastoma, ATRX processes cause synthetic lethality by MYCN amplification, helicase domain mutations cause DNA double-strand breaks, and multiple exon deletion leads to dysregulated protein expression and increased tumor aggressiveness. In PanNETs, ATRX promotes tumor progression by affecting downstream gene expression through combined mutations with IDH, MEN1. In osteosarcoma, ATRX mutations cause the formation of an ALT. All four tumors are closely related to the formation of ALT.

ATRX is known to function as a tumor suppressor, with mutations identified in many cancers (Pang et al., 2023). This summary highlights the impact of ATRX mutations on gliomas, neuroblastomas, pancreatic neuroendocrine tumors, and osteosarcomas. ATRX may be a potentially effective target for tumour therapy (Figure 4).

#### 2.5.1 Glioma

In astroglioma (Koschmann et al., 2016), IDH1^R132H^ combined with ATRX knockout induces APBs, the primary sites of *de novo* telomere synthesis in ALT-positive cells (Udugama et al., 2021). ATRX mutations occur in 50% of ALT-positive pediatric high-grade glioma (pHGG) patients (Stundon et al., 2023). Other pathways may drive ALT formation in various glioma types, but the mechanism linking ATRX loss to ALT remains to be further explored (Becher, 2023).

The combination of IDH1^R132H^/p53mut and ATRX loss induces immune escape by upregulating immune checkpoint proteins such as PD-L1, PD-L2, and the BET protein BRD3/4 in astrogliomas, as well as cytokines and chemokines such as IL-33, CXCL8, CSF2, IL-6, and CXCL9 (Hu et al., 2022).

Grade WHO II/III IDH-mutant gliomas, including oligodendrogliomas (IDH-O) with 1p/19q codeletion and astrocytomas (IDH-A) with intact 1p/19q, show differences in chromatin accessibility and gene expression that correlate with the extent of ATRX loss. IDH-A exhibits a greater incidence of ATRX loss, influencing the regulation of various chemokines, cytokines, and upstream transcription factors to sustain distinct tumor microenvironments (Babikir et al., 2021).

In most gliomas, ATRX deletion in combination with IDH mutation leads to immunosuppression, whereas IDH-mutant, ATRX-mutant astrocytomas exhibit greater immune cell infiltration than IDH-mutant, ATRX wild-type oligodendrogliomas (Venteicher et al., 2017). Studies indicate that ATRX deficiency alters the tumor microenvironment by inducing innate immune responses to dsRNA, upregulating chemokines such as CCL2, CCL5, CXCL10 and IFNβ in innate immune pathways, and enhancing immune CD4+Tcell infiltration (Hariharan et al., 2024). The contrasting immunological effects of ATRX deletion are attributed to the sequence and timing of ATRX mutations relative to IDH mutations, influencing distinct epigenetic alterations in chromatin.

In primary gliomas, DNA methylation downregulates ATRX, inhibiting DNA damage repair through the ATRX/EZH2 complex-PARP1 axis and increasing sensitivity to TMZ. Conversely, in TMZ-resistant glioma cells, DNA demethylation enhances ATRX expression, promoting DNA damage repair to counteract drug effects like TMZ (Han et al., 2020).

SIRT2, which was identified through multiomics synthesis as a potential driver gene in ATRX-null gliomas, is overexpressed. Inhibitors targeting SIRT2 induce epigenome reprogramming in ATRX-deficient glioma models by modifying H3K27ac and H4K16ac marks on chromatin and causing genome-wide changes via enhancer-associated modifications, promoting senescence in the ATRX-deficient glioma model system (Malgulwar et al., 2024).

#### 2.5.2 Neuroblastoma

According to genome aberrations, high-risk neuroblastomas can be divided into four categories: MYCN amplification (37%), TERT rearrangement (23%), lack of ATRX (11%), and not mutant (29%) (Brodeur, 2003; Cheung and Dyer, 2013; Zeineldin et al., 2020). Previous genomic analysis of stage 4 pediatric neuroblastoma samples identified ATRX mutations occurred among patients who were typically older than 5 years of age, had an indolent disease course, and poor overall survival (OS) (Cheung et al., 2012; Cheung and Dyer, 2013; Mossé et al., 2014).

However, MYCN expression and ATRX mutations are incompatible. Elevated MYCN levels promote metabolic reprogramming, mitochondrial dysfunction, reactive-oxygen species generation, and DNA-replicative stress. The combination of replicative stress caused by defects in the ATRX–histone chaperone complex, and that induced by MYCN-mediated metabolic reprogramming, leads to synthetic lethality (Zeineldin et al., 2020). Therefore, ATRX and MYCN represent an unusual example, where inactivation of a tumor-suppressor gene and activation of an oncogene are incompatible. This synthetic lethality may eventually be exploited to improve outcomes for patients with high-risk neuroblastoma.

ATRX aberrations were classified as nonsense mutations, missense mutations, or multiexon deletions (MEDs), with 68% identified as MEDs. Among these MEDs, 75% were predicted to be in-frame. The predominant deletion patterns involved the absence of exons 8-9 or exons 11-12 (Zeineldin et al., 2020).

Nonsense mutations in ATRX are scattered across the gene and can result in the absence of ATRX protein production; however, the impact of these mutations on neuroblastoma remains insufficiently studied (Zeineldin et al., 2020). Among these patients, those with ATRX missense mutations may have slightly better prognoses than those with the other two types of mutations (Zeineldin et al., 2020). It is unclear whether the missense mutation leads to reduced or missing expression of the neuroblastoma ATRX protein. Interestingly, in patients with ATRX syndrome, which is characterized by mild α-thalassemia symptoms and moderate intellectual disability, most missense mutations cause protein instability and increased susceptibility to degradation, resulting in reduced protein levels (Ratnakumar et al., 2012; Bieluszewska et al., 2022). ATRX mutations predominantly occur in the helicase domain, which is crucial for ATRX DNA translocation activity and can disrupt processes such as histone variant H3.3 incorporation and G-quadruplex or R-loop resolution (Zeineldin et al., 2020). In addition to point mutations and indels at the ATRX locus, large deletions at the N-terminus of ATRX can produce in-frame fusion proteins (IFFs). These IFFs lack critical chromatin interaction domains and contribute to aggressive neuroblastoma by altering chromatin structure and causing transcriptional dysregulation.

Some common chromosome aberrations in neuroblastoma, including recurrent distortions such as gain of 17q and loss of 11q, 2p, and 1p, are associated with poor prognosis (Brady et al., 2020). Loss of 11q occurs more frequently only in tumors lacking ATRX than in those with the other two mutation subtypes (Zeineldin et al., 2020). The region lost on 11q contains numerous genes involved in cell apoptosis and cell cycle progression. ATRX mutations are known to lead to increased replication-associated DNA damage. Therefore, the acquisition of 11q deletions may confer an evolutionary advantage when ATRX is abnormal, potentially reducing the level of DNA damage-induced apoptosis.

Almost all neuroblastoma patients with ATRX distortion suffer from ALT (Zeineldin et al., 2020; van Gerven et al., 2022). ATRX mutations contribute to tumorigenesis in two ways. First, they cause defects in telomeric H3.3 deposition, leading to telomere maintenance via ALT (Li et al., 2019). Second, ATRX mutations induce abnormal H3.3 deposition at gene promoters and enhancers involved in neuronal differentiation (including retinoic acid-responsive genes), resulting in structural defects at G4 sites. This attenuation of gene expression allows ATRX-mutated neuroblastoma cells to proliferate continuously (Zeineldin et al., 2020). Although DAXX mutations can also promote ALT, they do not affect the deposition of H3.3 at G4 structures.

#### 2.5.3 PanNETs

A total of 43% of PanNET patients had DAXX (25%) or ATRX (18%) mutations, and some exhibited ATRX loss (10%) (Heaphy and Singhi, 2023). The establishment of ATRX-KO mutants in pancreatic β cells via a genetically engineered mouse model did not directly cause PanNETs (Chan et al., 2018). However, simultaneous deletion of ATRX, MEN1, and PTEN resulted in the development of high-grade PanNETs in mice (Fuentes et al., 2024). The role of ATRX mutations in PanNETs is not well understood, but it is widely acknowledged that the simultaneous loss of multiple tumor suppressors is pivotal for PanNET development.

Additionally, DAXX/ATRX and ALT serve as prognostic biomarkers for PanNETs, with DAXX/ATRX mutations associated with a better prognosis. ALT-positive NF-PanNETs are significantly correlated with larger tumor size, increased perineural invasion, lymphovascular invasion, advanced pathological T stage and grade, regional lymph node metastases and postoperative distant metastasis/recurrence (*p* < 0.001) (Heaphy and Singhi, 2023).

ATRX mutations in PanNETs also influence gene expression through epigenetic mechanisms. Specifically, ATRX, DAXX, and MEN1 mutants (A-D-M mutant PanNETs) exhibit reduced histone methylation levels compared to wild-type PanNETs (Chan et al., 2018). As a critical transcription factor in pancreatic development and β-cell maturation, PDX1 exhibits hypermethylation and low expression of CpG sites in A-D-M mutant PanNETs, promoting a pancreatic “α-cell-like” phenotype thought to be the tumor’s origin (Chan et al., 2018).

#### 2.5.4 Osteosarcoma

Osteosarcoma is a rare and highly metastatic bone cancer originating from mesenchymal cells that predominantly affects children and adolescents (Lindsey et al., 2017). It is characterized by chromosomal instability and genetic alterations leading to aneuploidy and increased tumor aggressiveness (Leibowitz et al., 2015). In exome sequencing studies involving 1,244 osteosarcoma patients, ATRX was identified as a susceptibility gene, and patients with ATRX syndrome exhibited more aggressive forms of osteosarcoma (Mirabello et al., 2020).

Loss of ATRX in overall survival (OS) tumors has been reported to promote migration and invasion by upregulating NF-κB signaling and enhancing integrin binding (Bartholf DeWitt et al., 2022). In osteosarcoma, the ALT-positive phenotype, which is typically associated with ATRX mutation or loss of expression, is also observed in high-grade pediatric osteosarcoma, where telomere maintenance occurs despite wild-type ATRX expression. This is achieved through TOP3A amplification or overexpression, enabling ALT activation, overcoming replicative senescence, and promoting tumor progression (de Nonneville et al., 2022). The specific impact of ATRX mutation on osteosarcoma remains unstudied, but understanding this effect and the factors influencing ALT formation will be crucial for diagnosing and treating osteosarcoma.

## 3 Discussion

Studies have unequivocally established ATRX as a chromatin remodeler and a critical participant in various vital cellular processes.

ATRX syndrome manifests as multiple bodily system abnormalities (Badens et al., 2006; Gibbons et al., 2008; Dhayalan et al., 2011; Palaniappan and Ramalingam, 2017; Bieluszewska et al., 2022), which are related to various factors, such as ATRX structural alterations, interacting proteins, and the regulation of gene expression. ATRX syndrome primarily arises from ATRX mutations, which extensively disrupt neuronal cell development (Bérubé et al., 2005; Ritchie et al., 2008; Medina et al., 2009) and dysregulate α-globin transcription, mirroring the clinical manifestations observed in individuals with α-thalassemia (Ratnakumar et al., 2012; Li et al., 2016). The intellectual retardation and gonadal abnormalities displayed by the ATRX syndrome model constructed by knockout in different cells/tissues indicate that ATRX is widely involved in the development of the brain and gonads (León and Harley, 2021). Studies have shown that mutations in ATRX affect DNA repair by affecting cell cycle progression by affecting the abnormality of neuronal stem cells and gonad cell development (Bagheri-Fam et al., 2011; Han et al., 2020). Therefore, more research on the function of ATRX in chromosome segregation and the cell cycle is warranted. ATRX syndrome is closely related to mutations in ATRX, and its detection and risk of in carriers is particularly important because of its significant impact on child intelligence (León and Harley, 2021). However, the specific mechanism remains undefined, and the etiology of other systemic abnormalities remains under investigation.

Currently, studies have demonstrated that ATRX plays a significant role in tumorigenesis (Picketts et al., 1996; Danussi et al., 2018; Ohba et al., 2020; Gulve et al., 2022; Hackeng et al., 2022). Among the myriad factors implicated in tumor initiation and progression, abnormalities in ALT are notable features associated with ATRX dysregulation. Moreover, ATRX is involved in the development of cancers involving ALT, such as neuroblastoma, pancreatic neuroendocrine tumors and osteosarcoma (Olar and Sulman, 2015; Li et al., 2019). Nevertheless, the underlying causes of ALT remain to be elucidated through further studies.

G4s are predominantly observed in heterochromatic repeat regions, telomeres, pericentric repeat regions, rDNA repeats and endogenous retroviral elements (ERVs) (Law et al., 2010; Sarma et al., 2014; He et al., 2015; Sadic et al., 2015; Voon et al., 2015; Danussi et al., 2018; Udugama et al., 2018), which have adverse effects on gene expression, the progression of replication and the stability of DNA duplexes (Law et al., 2010; Clynes et al., 2015; Teng et al., 2021). ATRX prevents G4 structure formation by maintaining heterochromatin in G4-rich regions through DAXX-mediated incorporation of variant H3.3. The condensation of heterochromatin is crucial for protecting cells from the harmful effects of G4-induced stress (Clynes et al., 2014; Teng et al., 2021). In addition, ATRX modulates gene expression not only directly but also in collaboration with other proteins (Medina et al., 2009; Law et al., 2010; Clynes et al., 2014; Chu et al., 2017; Yan et al., 2022). This action prevents further double-strand breaks and ALT formation, thereby reducing cancer occurrence (Law et al., 2010; Leung et al., 2013; Clynes et al., 2015; Ramamoorthy and Smith, 2015; Nguyen et al., 2017). However, the specific etiological factors underlying ALT levels are unknown and require further investigation.

The MRN and MCM proteins discussed in this paper represent only a fraction of the many proteins implicated in the mechanism of ALT. However, the specific mechanism by which these proteins interact with ALT remains to be further studied. It has also been reported that the overexpression of Sp100, another PML nucleosome component, can inhibit ALT by separating MRN components from APB (Jiang et al., 2005). Hence, ATRX expression could similarly influence Sp100 overexpression, limiting ALT by relocating the MRN complex from the telomeric recombination site. Notably, the MRN complex favors G4 structures as its substrate (Ghosal and Muniyappa, 2005), potentially directly cleaving persistent G4 structures in ALT tumors after DNA replication or transcription, leading to DSB formation and HDR (Seah et al., 2008). The mechanism involved in the interaction between ATRX and MCM through H3.3 remains unclear.

Studies have shown that ALT can develop in the absence of ATRX mutations, rendering ATRX-targeted therapies ineffective for some ALT-positive gliomas, necessitating alternative treatments (Stundon et al., 2023). Additional ALT mechanisms may exist but have yet been investigated.

In glioma, ATRX/IDH/p53 combined mutations and individual mutations exhibit opposite effects on the immune microenvironment, which encourages tumor proliferation and invasion (Hu et al., 2022; Hariharan et al., 2024). Our hypothesis is that these mutations cooperate to affect chemokine and signal transducer expression and, consequently, the tumor microenvironment. As a therapeutic drug for glioma, TMZ inhibits tumor cell proliferation mainly by inhibiting DNA damage repair in ATRX mutation-positive glioma (Han et al., 2020). The resistant strain maintains DNA repair by altering ATRX transcription levels through demethylation of its transcription factors (Han et al., 2020). Studies suggest that TMZ + γ-radiotherapy enhances the immunosuppressive effects in the absence of ATRX, inducing an immunosuppressive microenvironment (Hu et al., 2022). This proposes modifying the demethylation levels of glioma chemoradiotherapy or combining it with immunomodulatory medications to improve efficacy.

As a transcription factor, ATRX plays a broad role in epigenetic development (Haase et al., 2018). It controls gene transcription via histone modifications, promoting tumor initiation and progression, while its own post-translational modifications (PTMs) are undefined (Delbarre et al., 2017; Malgulwar et al., 2024). Studies report that phosphorylation at multiple sites of ATRX is strongly related its role in the cell cycle (Bérubé et al., 2000). The impact of the modification of ATRX itself on its function remains to be thoroughly investigated.

Through their effects on the immune microenvironment and cytokine production, IDH and ATRX mutations in glioblastoma and astrocytoma promote immune suppression, and may be a potential therapeutic target for prolonging patient survival (Hu et al., 2022; Hariharan et al., 2024; Squalli Houssaini et al., 2024). In addition, in pancreatic neuroendocrine tumors, ATRX/DAXX mutations are considered prognostic biomarkers in patients with poor disease-free survival (DFS), but opposite results in advanced/metastatic patients (Wang et al., 2021), indicating that ATRX mutations at different stages may have different effects and have implications for prognostic judgment. With the development of medicine, how ATRX mediates ALT, affects immune functions, epigenetic and its role in tumor development will be studied in the near future. ATRX opens avenues for discovering novel precision medicine-based therapeutic approaches.
